# The Texas Health Officers’ Association

**Published:** 1908-06

**Authors:** L. B. Bibb

**Affiliations:** Secretary


### TEXAS MEDICAL JOURNAL.

Established July, 1885.

F. E. DANIEL, M. D., - - - - Editor, Publisher and Proprietor.

PUBLISHED MONTHLY.—SUBSCRIPTION $1.00 A YEAR.

VOL. XXIII. AUSTIN, JUNE, 1908. No. 12.

The publisher is not responsible for the views of contributors.

### Department of Public Health.

### The Texas Health Officers' Association.

Organized at Corpus Christi, Texas, May 15, 1908.

OFFICIAL REPORT OF THE SECRETARY.

Published exclusively in the TEXAS MEDICAL JOURNAL, and reported by
L. B. Bibb, M. D., Secretary Department of Public Health, Secretary.
W. M. Brumby, M. D., State Health Officer, President.

MINUTES OF MEETING.

CORPUS CHRISTI, TEXAS, May 15, 1908.

Pursuant to call of State Health Officer W. M. Brumby, a large number of physicians in attendance upon the meeting of the Texas State Medical Association, principally county and city health officers, representing a large area of the State, met in Elks' Hall and effected an organization by the election of a President, a Secretary and sixteen Vice-Presidents, one for each congressional district of the State, and the adoption of a Constitution and By-Laws.

Dr. W. M. Brumby, presiding, stated the objects and purposes of the organization to be the better protection of the public health by the enforcement of measures for the prevention and removal of causes operating to cause sickness and death; a co-operation of local health officers with the State officials and with the National health authorities, and a uniform system of vital statistics, inspections and reports.

Dr. Brumby was unanimously elected President. Dr. L. B. Bibb was unanimously elected Secretary. A committee (McGregor, Laredo; Terrill, Galveston, and Young, Austin) was appointed to nominate sixteen local health officers, one from each congressional district represented at the meeting, to be Vice-Presidents, and they reported the following:

District No. 1.—Dr. D. J. Jenkins........Daingerfeld, Texas.
District No. 2.—Dr. Holman Taylor..........Marshall, Texas.
District No. 3.—Dr. B. J. Hubbard..........Kaufman, Texas.
District No. 4.—Dr. W. C. Bryant..........McKinney, Texas.
District No. 5.—Dr. J. H. Florence............Dallas, Texas.
District No. 6.—Dr. W. C. Blalock.............Kosse, Texas.
District No. 7.—Dr. E. B. Parsons..........Palestine, Texas.
District No. 8.—Dr. J. W. Scott.............Houston, Texas.
District No. 9.—Dr. J. M. Andrews..........Wharton, Texas.
District No. 10.—Dr. E. M. Thomas........Georgetown, Texas.
District No. 11.—Dr. Marcus Smart..............Eddy, Texas.
District No. 12.—Dr. R. B. West...........Fort Worth, Texas.
District No. 13.—Dr. J. S. Wilkins.........Wellington, Texas.
District No. 14.—Dr. L. G. Wille........New Braunfels, Texas.
District No. 15.—Dr. W. W. McGregor.........Laredo, Texas.
District No. 16.—Dr. J. W. Blaisdell.........Ballinger, Texas.

The report was adopted and the committee discharged.

The question of electing a Treasurer was discussed, and it was finally agreed that there should be no annual dues or assessments, and no Treasurer was necessary and none was elected.

Every health officer present, whether county or city, reported upon the sanitary condition of his locality, what he and the authorities have done and are doing. [These reports will be published *in extenso* in July number of TEXAS MEDICAL JOURNAL.]

The committee appointed to draft a Constitution and By-Laws submitted the following, which, after reading section by section, was adopted. The committee consisted of Dr. E. B. Parsons, of Palestine; Dr. J. M. Andrews, of Wharton, and Dr. J. W. Blaisdell, of Ballinger.

### CONSTITUTION AND BY-LAWS OF THE TEXAS HEALTH OFFICERS' ASSOCIATION.

### *Article I.—Name of the Association.*

The name and title of this Association shall be the Texas Health Officers' Association.

### *Article II.—Purposes of the Association.*

The purposes of the Association shall be to bring into touch with each other all the sanitarians and constituted health authorities within the confines of Texas, with a view to mutual aid in time of pestilence and emergency; to wider dissemination of knowledge of hygience among the citizens of the State; to further improvement of our local and general health laws, and to more efficient co-operation in the crusade against all communicable and preventable diseases.

*Article III.—Composition of the Association.*

Section 1. This Association shall consist of members, delegates and guests.

Sec. 2. The members of this Association shall be legally qualified city and county health officers, members of local boards of health, either appointed or elected, members of boards of health of unincorporated towns, sanitarians and others interested in the promotion of public health.

Sec. 3. Guests shall be such distinguished sanitarians or hygienists from without the State who being present are invited to take part in the scientific work of any given session of the Association. Every city, incorporated town, business league or association in the State shall be entitled to one delegate each.

*Article IV.—Sessions and Meetings.*

Section 1. The Association shall hold an annual session which shall be open to all registered members and guests.

Sec. 2. The time and place for holding each annual session shall be fixed by the Central Executive Committee.

*Article V.—Officers.*

Section 1. The officers of this Association shall be a President, who shall be the State Health Officer, sixteen Vice-Presidents, and a Secretary-Treasurer.

Sec. 2. These officers, except the President, shall be elected for a term of one year, and shall serve until their successors are duly installed, and in case of the death or resignation of any Vice-President, or the Secretary-Treasurer, the vacancy shall be filled by the Central Executive Committee on nomination by the President.

Sec. 3. The officers of the Association shall be elected by the Association at the close of the annual session, and no person shall be elected to any office who shall not have been a member of the Association since its organization, or at least two years.

*Article VI.—The Central Executive Committee.*

Section 1. The sixteen Vice-Presidents, the President and the Secretary-Treasurer shall constitute the Central Executive Committee.

Sec. 2. The Vice-Presidents shall be chosen one from each congressional district, with special reference to his fitness, previous experience and qualifications in matters pertaining to the public health.

Sec. 3. The Central Executive Committee shall transact any and all business that comes up between sessions, shall arrange the program for the reading of papers at each session, and shall have general supervision of the affairs of the Association.

Sec. 4. The Central Executive Committee shall have authority by a two-thirds vote to pass any measure over the veto of the President.

BY-LAWS.

*Article I.—Membership.*

Section 1. The statement of an applicant that he is a properly and legally qualified health officer in any locality in this State shall be sufficient to admit him to membership, and any objections as to his eligibility must be presented to the Central Executive Committee, and if a majority of same favor his admission he shall be a member.

Sec. 2. Eeach member in attendance at the annual session shall enter his name on the registration book, indicating the city and county which he resides in, and shall receive a badge, which shall be evidence of his right to all the privileges of membership at that session.

*Article II.—Annual and Special Sessions of the Association.*

Section 1. The Association shall hold an annual session at such time and place as shall have been fixed at the preceding annual session.

Sec. 2. Special sessions of the Association shall be called by the President at his discretion, or on petition of nine of the members of the Central Executive Committee.

*Article III.—General Meetings.*

Section 1. The general meeting shall include all registered members and guests, and all shall have equal rights to participate in the proceedings and discussions and, except the guests, to vote on pending questions. Each general meeting shall be presided over by the President, or in his absence or disability, or by his request, by one of the Vice-Presidents. Before it, and at any such time and place as may have been arranged, shall be delivered the annual address of the President and the annual oration, and the entire time of the session, so far as may be, shall be devoted to papers and discussions relating to scientific sanitation.

Sec. 2. The general meeting shall have authority to create committees or commissions for scientific investigation of special interest and importance to the Association or the public, and to receive and dispose of same.

Sec. 3. Except by special vote, the order of exercises, papers and discussions as set forth in the official program shall be followed from day to day until it has been completed.

Sec. 4. No address or paper before the Association, except those of the President and orators, shall occupy more than twenty minutes in its delivery, and no member shall speak more than five minutes nor more than once on one subject.

Sec. 5. All papers read before the Association shall be its property. Each paper shall be deposited with the Secretary when read.

*Article IV.—Central Executive Committee.*

Section 1. A majority of the members of the Committee shall constitute a quorum, and all of the meetings of the Committee shall be open to members of the Association.

Sec. 2. Each member shall give diligent attention and foster the scientific work and spirit of the Association, and shall constantly study and strive to make each annual session a stepping stone to future ones of higher interest.

Sec. 3. It shall consider and advise as to the material interests of the Association and of the public in those important matters wherein it is dependent upon the Association, and shall use its influence to secure and enforce all proper medical and public health legislation, and to diffuse popular information in regard thereto.

Sec. 4. It shall make careful inquiry into the condition of the public health in each county in the State, and each member shall keep in touch with the State Health Officer and be prepared to assist him in organizing the health officers of his district with a view to securing concerted action against all pestilential diseases that may visit his district. Also each member shall keep well in touch with local conditions in his district and with the various health officers therein, so as to stimulate interest in the promotion of public health.

Sec. 5. It shall have authority to appoint committees for special purposes from among members of the Association who are not members of the Central Committee, and such committees may report to the Central Executive Committee and participate in the discussion thereon.

*Article V.—Duties of Officers.*

Section 1. The President shall preside at all meetings of the Association and of the Central Executive Committee; shall appoint all committees not otherwise provided, and serve as *ex officio* Chairman of same; shall deliver an annual address at such time as shall be arranged; shall give a deciding vote in case of a tie, and shall perform such other duties as parliamentary usage may require. He shall have the power and authority to veto any measure passed by the Association or the Central Executive Committee, but a two-thirds vote of either body shall override his veto. He shall, as far as practicable, visit the various sections of the State and assist the members in building up favorable sentiment towards sanitation, and in making their work more practicable and useful.

Sec. 2. The Vice-Presidents shall assist the President in the discharge of his duties, and, in addition, shall serve as members of the Central Executive Committee, as set forth hereinbefore.

Sec. 3. The Secretary-Treasurer acting with the Committee on Scientific Work, shall prepare and issue the programs for and attend all meetings of the Association, and he shall also attend the meetings of the Central Executive Committee, of which he is *ex-officio* member, and shall keep minutes of the Association and of the Committee in separate record books. He shall be custodian of all record books and papers belonging to the Association, and he shall provide for the registration of members at annual sessions, and also keep a card index of the members of the Association. In so far as it is in his power, he shall use the printed matter, correspondence and influence of his office to aid the Central Committee in the organization, and improvement of the Association, and in the extension of its power and usefulness. He shall conduct the official correspondence of the Association, notifying members of meetings, officers of their election, and committees of their appointment and duties. He shall act as Chairman of the Committees on Scientific Sanitation and on Publication, and shall liberate monthly popular information for the lay press such as is calculated to build up public sentiment in the direction of improving the public health.

*Article VI.—Committees.*

Section 1. The Committee on Scientific Work shall consist of three members, of which the Secretary shall be one and chairman, and shall determine the character and scope of the scientific proceedings of the Association for each session, subject to the instructions of the Association or the Central Executive Committee, or to the provisions of the By-Laws and Constitution. Previous to each annual session it shall prepare and issue a program announcing the order in which papers, discussions and other business shall be presented, which shall be adhered to by the Association as nearly as practicable.

Sec. 2. The Committee on Public Policy and Legislation shall consist of three members of the Central Executive Committee, the President and Secretary. Under the direction of the Association and the Central Executive Committee it shall represent the Association in securing and enforcing legislation in the interest of the public health.

*Article VII.—Rules of Order.*

The deliberations of this Association shall be governed by parliamentary usage as contained in Roberts' Rules of Order, unless otherwise determined by vote.

---

The following papers were read:

*Commonsense Scientific Quarantine Methods,* Dr. C. W. Trueheart, City Physician, Galveston.

*Sanitation of Meat Markets,* Dr. B. T. Young, Assistant State Health Officer, Austin, Texas.

*Needed Reforms in Our Laws on Insanity,* Dr. Frank Ross, Assistant City Physician, Houston.

*The Compensation of the County Health Officer,* Dr. H. F. McCoy, La Porte, Texas.

*Elements of Decay in Our National Life* (read by title), Dr. F. E. Daniel, Austin, Texas.

*Bovine Tuberculosis* (read by title), Dr. W. J. Mathews, President City Board of Health, Austin, Texas.

That of Dr. Trueheart appears in this issue. The others will be published exclusively in the "Red Back" in July, August and September numbers.

The following resolutions were adopted:

MEMORIAL TO THE TEXAS STATE LEGISLATURE.

"WHEREAS, It is an established fact that tuberculosis in its many forms and stages is found among the inmates of the State institutions and little effort at segregation is possible to be made whereby the unfortunate victims might be benefited by appropriate treatment or prevent their becoming a menace to the health of others, and

"WHEREAS, The disease is fast becoming known as a poor man's disease because of his inability to procure proper treament, diet and climate, and because of his inability to protect his family against the infection—partly from his own environments and partly to his gradually becoming inured to his disease and to his condition in life—want of hope and lack of intelligent training.

"WHEREAS, Our climate, pure atmosphere and maximum of sunshiny days is attracting tubercular invalids from other States, and many are in indigent circumstances and a charge on the charities of an already burdened community—a further menace.

"WHEREAS, Many other States have already awakened to a sense of their responsibility, and have provided sanitoria for their indigent tubercular, thereby lessening the danger of infection to others, and urging Legislatures and philanthropists to lend aid to those unable to assist themselves, and

"WHEREAS, Believing as we do that all contagious and infectious diseases are preventable and amenable to treatment, and, furthermore, that it is incumbent upon a prosperous and enlightened State like this to care for its unfortunates, not only from a humane standpoint, but with a view of protection to others, and

"WHEREAS, To sum up in a word the sanatorium

"1. Relieves the State of its present responsibility in caring for its charges who by force of circumstances are penned up together to contract diseases possible to prevent.

"2. It offers the invalid the most practical method which under favorable conditions would cure or arrest disease and at the same time acquire hygienic education in its prevention.

"3. It relieves the community of one more nidus of infection.

"4. A valuable educational factor in the care and prevention of the disease.

"5. A definite and important place for scientific study and investigation of the disease and its complications, which can scarcely otherwise be obtained. Therefore, be it

"*Resolved*, That the members of the next Legislature separately and collectively be requested to make an appropriation of not less than $100,000 for the construction, establishment and maintenance of a sanatorium for the care and treatment of the State's tubercular patients. Be it further

"*Resolved,* That in order that an influx of such immigration from other States does not occur and fill such institution that one of the requirements for entrance be that the applicant shall have been a *bona fide* resident of this State for a period of three years; and be it further

"*Resolved,* That this memorial be presented to the next Legislature by a committee from this body appointed by the Central Executive Committee of this Association."

---

The following is a list of members of the Texas Health Officers' Association signing the register at the first meeting:

LIST OF MEMBERS.

Stephen M. Jordan, County Health Officer, Liberty, Liberty county.

M. P. Smart, City Health Officer, Eddy.

G. P. Day, City and County Health Officer, Madisonville, Madison county.

Benj. F. Gibson, Prison Physician, Huntsville.

M. F. Cox, County Health Officer, Canton, Van Zandt county.

L. B. Bibb, Quarantine Officer, Pass Cavallo.

B. T. Young, Assistant State Health Officer, Austin.

C. L. McClellan, City Health Officer, Rockport.

F. A. Pierce, County Health Officer, Ferris, Ellis county.

E. B. Parsons, County Health Officer, Palestine, Anderson county.

T. W. Moore, City and County Health Officer, Seguin, Guadalupe county.

J. H. Florence, Quarantine Officer, Galveston.

D. M. Stewart, County Health Officer, Canyon City, Randall county.

J. W. Blaisdell, City and County Health Officer, Ballinger, Runnels county.

Oscar D. Rodney, City Health Officer, Mt. Calm, Hill county.

Albert H. Mitchie, City and County Health Officer, Childress, Childress county.

Wm. Veazey, City Health Officer, Van Alstyne.

A. C. Cates, Correspondent. General Sanitarian, etc., Crowell.

J. O. Brockman, County Health Officer, Brackenridge, Stephens county.

J. M. Still, County Health Officer, Kemp, Kaufman county.

W. A. Wills, City Health Officer, Lancaster.

L. G. Wille, City Health Officer, New Braunfels.

R. B. West, County Health Officer, Fort Worth, Tarrant county.

H. J. Hamilton, U. S. P. H. and M. H. S., Laredo.

J. G. Coleman, City and County Health Officer, Coleman, Coleman county.

B. A. Swinney, County Health Officer, Newton, Newton county.

J. M. Andrews, County Health Officer, Wharton, Wharton county.

A. B. Gardner, City Health Officer, Denison.

B. J. Hubbard, City Health Officer, Kaufman.

W. W. Magregor, City and County Health Officer, Laredo, Webb county.

J. F. Corry, County Health Officer, Rockwall, Rockwall county.

R. C. Hall, City Health Officer, Marshall.

R. W. Skipper, City Health Officer, Lovelady.

J. D. Stocking, County Health Officer, Clarendon, Donley county.

W. A. Warner, County Health Officer, Claude, Armstrong county.

W. E. Lowry, Quarantine Officer, Laredo.

T. J. Turpin, County Health Officer, Corpus Christi, Nueces county.

W. C. Bryant, County Health Officer, McKinney, Collin county.

W. C. Blalock, County Health Officer, Kosse, Limestone county.

E. S. Cox, County Health Officer, Galveston, Galveston county.

J. Holman Taylor, County Health Officer, Marshall, Harrison county.

J. E. Gibson, City Health Officer, McKinney.

J. G. Smith, Quarantine Officer, Velasco.

C. H. Reagan, County Health Officer, Oakville, Live Oak county.

E. M. Thomas, County Health Officer, Georgetown, Williamson county.

S. B. Maxey, County Health Officer, Angleton, Brazoria county.

H. Leonards, County Health Officer, New Braunfels, Comal county.

E. E. Ledbetter, City Health Officer, Tioga.

J. S. Wilkins, County Health Officer, Wellington, Collingsworth county.

T. Shearer, County Health Officer, Wallisville, Chambers county.

J. B. DuBose, Assistant County Health Officer, Humble, Harris county.

M. L. Gunn, County Health Officer, Miami, Roberts county.

W. M. Brumby, State Health Officer, Austin.

James J. Terrill, Correspondent, General Sanitarian, etc., Galveston.

H. F. McCoy, City Health Officer, La Porte.

J. H. McCracken, County Health Officer, Mineral Wells, Palo Pinto county.

L. Coons, County Health Officer, Wichita Falls, Wichita county.

L. S. Johnston, City Health Officer, Atlanta.

E. L. Sharpe, County Health Officer, Pleasanton, Atascosa county.

M. J. Phenix, County Health Officer, Colorado, Mitchell county.

F. E. Daniel, Correspondent, General Sanitarian, etc., Austin.

A. C. Cates, County Health Officer, Crowell, Foard county.

D. J. Jenkins, County Health Officer, Daingerfield, Morris county.

C. W. Trueheart, City Health Officer, Galveston.

C. O. Bryan, City and County Health Officer, Center, Shelby county.

J. W. Hargus, County Health Officer, Cotulla, La Salle county.

N. W. Gustine, County Health Officer, Barstow, Ward county.

J. A. T. Page, Correspondent, General Sanitarian, etc., Falfurrias, Starr county.

J. O. Matthews, City Health Officer, Howe.

P. J. Shaver, Quarantine Officer, El Paso.

Jas. A. Hill, County Health Officer, Groveton, Trinity county.

F. B. Seymour, County Health Officer, Beeville, Bee county.

Sig Burg, City Health Officer, San Antonio.

D. Berry, County Health Officer, San Antonio, Bexar county.

Ray B. Wright, County Health Officer, Carrizo Springs, Dimmit county.

E. A. Johnson, Correspondent, General Sanitarian, etc., Amarillo.

The meeting adjourned to meet again at Galveston in May, 1909, two days prior to the annual convention of the State Medical Association, and subject to call of the President *ad interim*.

L. B. BIBB, M. D., Secretary.

The following paper was read and ordered published in the "Red Back":

### Commonsense Scientific Quarantine Methods.

BY C. W. TRUEHEART, M. D., GALVESTON.

There are quarantine methods held to be in accordance with common sense, but lacking in scientific demonstration and accuracy, and methods passing for scientific that are sadly at variance with common sense.

Certain well-established facts, if rightly considered and duly weighed, can scarcely fail to materially modify the views of intelligent observers as to the safety, necessity and expediency of material changes in prevailing quarantine methods—changes that would work valuable relaxation in some vexatious, costly and really unnecessary obstructions and restrictions now imposed upon commerce at Texas ports.

To justly appreciate the correctness of this proposition and the legitimacy and force of the conclusions sought to be established in this paper, the following facts should be steadily borne in mind:

1. That the female Stegomyia fasciata mosquito, when infected by biting a yellow fever patient within seventy to one hundred and twenty hours after the inception of the disease, and after the lapse of eleven to eighteen days, the period required for the yellow fever materies morbi to undergo in the mosquito certain developmental changes is, absolutely and solely, the only agency by which the disease can by any possibility be conveyed.

2. That that pernicious old notion that yellow fever is liable to be carried by fomites, or to be communicated to a non-immune by contact with a case of the fever has been conclusively disproved.

3. That about three days after the inoculation of a non-immune person by the bite of the infected female Stegomyia fasciata mosquito is the usual time for the disease to develop.

4. That the elaborate and extended series of most scientifically and carefully conducted, practical experiments, carried out in the vicinity of Havana in 1902 by the lamented Walter Reed and his able co-workers; and since then by Surgeon Gorgas and numerous other investigators in different localities, have all demonstrated, beyond the question of a doubt, the absolute correctness of "the mosquito theory"; indeed, it is a theory no longer, but one of the most thoroughly well-demonstrated and reliable facts in the whole realm of modern scientific investigation and discovery.

5. That the complete and systematically maintained extermination of the Stegomyia fasciata mosquito really constitutes the whole gist, the *sine qua non,* in dealing with the vexed yellow fever question and in the carrying out of efficient quarantine measures for the protection of the public with least interruption and injury to commerce, maritime or inland. That with this species of mosquito extinct the dreaded disease is robbed of its dangers and its terrors.

6. That this mosquito, being domestic in its habits, just as the barnyard fowl is a domestic bird, its breeding place not being in marshes, but almost exclusively in the vicinity of human habitations; unlike the Culex variety, is not migratory, but sticks to the locality where hatched, this all conduces to rendering it more ready for access and extermination than other species. That its extermination can be achieved and maintained and yellow fever thereby banished from the locality is a fact that has been demonstrated time and again. In Havana, for instance, where the disease has held sway the greater part of every year for one hundred and fifty years uninterruptedly, by virtue of mosquito extermination during the several years that the United States military authorities had control of sanitary affairs there, the fever was entirely stamped out, and that mosquito being extinct cases of the disease introduced from other places were cared for without spread of the malady. Similar results have also been secured by numerous other sanitarians in other localities.

7. In Galveston, too, by dint of cistern screening, treating with crude carbolic acid all cesspools in the unsewered districts, all containers of water held on the dock fronts, cotton presses, yards, etc., for fire extinquishing purposes, the yellow fever spreading mosquito has been practically so completely exterminated that numerous instances have occurred during the past three or four years when cases of the fever introduced here have been conveyed through the city in screened ambulance and treated behind screens at hospital; in hospital these patients were cared for by non-immune physicians and nurses and the medical students freely admitted to bedside clinics, and that, too, without spread of the disease or creating anxiety or alarm among the people.

In short, it has been demonstrated that the established and systematically maintained extermination of the yellow fever spreading mosquito is the only measure really required to enable sanitary authorities to safely deal with yellow fever on shipboard or on shore. It stands to reason, too, that Galveston or any other port which has ridded itself of that mosquito is, in common fairness entitled to be extempted from quarantine restrictions demanded where such mosquito extermination has not been effected.

It is self-evident, I contend, that at Galveston or any other port where this mosquito is extinct it is really unnecessary and an inexcusable hardship to detain at quarantine grounds ships from south of the 25th degree of north latitude, or from places suspected of or even known to be infected with yellow fever longer than is required to carry out quarantine measures on the following plan:

Immediately on the arrival of such ships, crew and passengers to be mustered and while the ship's manifest is being verified, temperatures, etc., taken cognizance of, all compartments of the vessel to be promptly and thoroughly fumigated so as to effectually destroy all mosquitoes aboard. Any person with suspicious symptoms to be cared for in a screend quarantine hospital, all the passengers to be held in quarantine detention quarters for six days from the completion of the fumigation. But the ship, with a trusted guard aboard to prevent the crew from leaving the vessel during the six days, to be allowed to proceed to the wharf without delay and discharge and take on cargo, etc. During this time the temperatures of the crew and passengers to be taken twice daily and all hands kept under close surveillance of a quarantine physician. Should cases of the disease occur on the ship they could be transferred in a screened compartment on the quarantine launch to the quarantine hospital, or in a screened ambulance, as has been done many times in the past four years, to one of the shore hospitals.

In case of steamers laden with fruit, or other perishable cargo, where the vessel has been reliably fumigated at port of departure, the same plan could be observed, except the omission of the fumigation on arrival.

This could all be carried out without creating alarm among the people and without danger of spreading the disease. With this plan faithfully observed as to passengers and crew there would be no danger of communicating the disease to the people of the port or to interior localities, even though the latter should be lax in the extermination of the mosquito.

To carry out such a quarantine, of course, detention quarters and an up to date hospital building would have to be provided at State quarantine stations. It can not be denied that without such equipment at the stations our Texas quarantines are a veritable travesty on up to date civilized methods of dealing with that all-important governmental function, the preservation of the public health and the protection of life, liberty and property.

The site of the present dilapidated old shack of a quarantine building at Galveston is a reproach and disgrace to our State; having scanty space and hemmed in as it is on all sides by Federal belongings and easily accessible to all comers, it is quite impossible of that isolation so necessary to a quarantine station and is, in point of location and equipment, quite unsuited to the needs of Galveston Pass, the great maritime gateway to Texas.

The north side of the channel, or on Bird Island Reef in Bolivar Roads, is really the logical location for the quarantine station.

The wonder is that our State Health Officers and local quarantine officers have been able to do as well as they have with such utterly inadequate equipment and facilities. It will certainly be a great oversight if the next Legislature fails, as did former Legislatures, to make adequate appropriations for quarantine purposes.

If Texas can not afford, or is not willing to conduct quarantine at her seaports on a plane in keeping with the vital importance of the interests involved, then the work should be turned over to the United States Marine Hospital Department. The State, in that case, maintaining only quarantine observation stations at its seaports.

As to quarantine charges, the Federal quarantine authorities exact no charge for inspections or fumigations. The great State of Texas ought certainly to be able to do as much for protecting the health and lives of her people and fostering their commercial transactions. It is a measure of public protection. The benefits are to our people, not to the ship owners.

Because such exactions are imposed by other States, European or American, is no just reason for Texas doing it. It is a stupid relic of barbarism and should be abolished at once and forever by an enlightened people.

The State should certainly make no charge for inspection and if anything for fumigation only the wholesale price of the stuff used should be required.

The notions (as I once heard a prominent man express it) "that those quarantine charges come out of the coffers of the bloated ship owners" is erroneous. The broker who charters the ship puts up the money, it is true, but it ultimately comes out of the pockets of our own people. In case of the ship bringing in imports, the consumer pays them; with the ship carrying exports, the farmers and other producers pay the same. In other words, these quarantine charges are simply absorbed into the ocean freight rates, which, of course, are in the end paid by our own people.

